# Effectiveness of social and therapeutic horticulture for reducing symptoms of depression and anxiety: a systematic review and meta-analysis

**DOI:** 10.3389/fpsyt.2024.1507354

**Published:** 2025-01-16

**Authors:** Carly J. Wood, Jo Barton, Claire L. Wicks

**Affiliations:** ^1^ School of Sport, Rehabilitation and Exercise Sciences, University of Essex, Colchester, United Kingdom; ^2^ School of Health and Social Care, University of Essex, Colchester, United Kingdom

**Keywords:** nature-based interventions, mental health, mental ill-health, mental illness, gardening, therapeutic horticulture

## Abstract

**Background:**

Depression and anxiety are the two most common mental health conditions, that often co-exist and relapse over time. There is a need for sustainable treatment options to address increasing rates of depression and anxiety and reduce the strain on health systems. Social and Therapeutic Horticulture (STH) is a nature-based health intervention that can reduce symptoms of depression and anxiety, however synthesised evidence of its effectiveness is required to inform commissioning and availability of interventions.

**Aim:**

The aim of this systematic review and meta-analysis was to examine the effectiveness of STH for reducing symptoms of depression and anxiety.

**Method:**

Following PRISMA guidelines and the protocol registered on Prospero (CRD42024542671) a systematic search of scientific databases and grey literature was conducted to identify quantitative studies examining the impact of STH interventions on depression and anxiety outcomes. Studies were eligible for inclusion if they reported on an STH intervention that was led by a trained practitioner, administered to adult populations who were identified as either at risk, with symptoms or diagnosis of depression and/or anxiety and reported on depression or anxiety outcomes measured using validated scales. Data from eligible studies were extracted and analysed using a random effects meta-analysis.

**Results:**

Seventeen studies were identified for inclusion including four RCTs, ten quasi-experimental studies with comparators and three single group studies. Nine studies reported outcomes for depression only, two for anxiety only and six for both depression and anxiety. Across studies with comparators, meta-analyses revealed large and significant effects in favour of the STH group for depression (SMD= -1.01; p=<.001) and moderate and significant effects in favour of the STH group for anxiety (SMD=-.62; p<.001). There was significant heterogeneity across studies, with high risk of bias, particularly for statistical validity and outcome measure reliability.

**Conclusions:**

STH interventions have the potential to support existing treatment approaches for depression and anxiety. However, to inform commissioning and upscaling of services, rigorous randomised studies comparing STH to treatment as usual for depression and anxiety are required.

## Introduction

Depression and anxiety are the two most common mental health conditions that are often co-morbid (1). Depression accounts for 4.3% of the global burden of disease (2), affecting approximately 280 million people (3), whilst anxiety affects 301 million people worldwide (4). In England 1 in 6 individuals aged 16+years experience symptoms of depression or anxiety in any given week (5), with rates continuing to increase (6). In 2021/22 referrals to the National Health Service (NHS) Talking Therapies (TTs) rose 22.2% from 1.44 million in 2017/18 to 1.76 million (7), whilst anti-depressant use increased by 164.6% between 2006 and 2022/23 (8). However, a diagnosis of depression or anxiety does not always result in appropriate or effective treatments. Up to 60% of patients prescribed anti-depressants do not adhere, and in 2022/23 only 49.9% of those who engaged in TTs were deemed ‘recovered’ (8). With depression and anxiety commonly relapsing, there is an increased likelihood of the need for repeated treatment. In England, over the next 3-5 years approximately 10 million people will require mental health support, with pre-existing conditions accounting for 66% of this provision (9). This level of demand is 2-3 times NHS capacity, with 1.2 million people currently on waiting lists for support (10). Thus, there is a need for sustainable treatment options to address the widening treatment gap.

Nature-based interventions (NBIs, e.g. fishing, gardening) are defined as programs, activities, or strategies that utilise nature to improve health and well-being (11). NBIs are increasingly used to prevent and treat mental ill-health, in line with the NHS and the UK Government commitment to the use of NBIs (12), the Government’s community-based mental health model (13) and a whole-systems approach to healthcare (14). Social and Therapeutic Horticulture (STH), also termed therapeutic horticulture (TH), is a specific type of NBI, where trained practitioners work with plants and people to improve an individual’s physical and psychological health, communication and thinking skills (15). Although used interchangeably with terms like horticultural therapy (HT), there are some differences between provisions which enable them to cater for varying levels of mental health needs (16). For example, STH or TH is appropriate for individuals with mild mental ill-health who need support from their GP and access to psychological therapies, medication and/or ongoing intervention. These individuals will likely need support to attend and will be working towards identified recovery goals with support from trained staff (16). Enhanced STH is designed for individuals with moderate mental ill-health who have more complex needs, will need additional support to attend, and a planned recovery pathway to enable them to move to the less supported STH provision. Finally, HT, is for individuals with complex or severe mental ill-health, who may be in acute crisis or have a long-term condition that affects daily function. These individuals may access activities within a hospital setting, will need continuous support and a recovery pathway into enhanced STH provision (16). Whilst the level of mental health need and support varies across provision, a key requirement of any level of STH intervention is the delivery by a trained practitioner who can tailor the gardening activities to individual needs, preferences, and recovery goals, making it distinct from community, social or self-directed gardening.

Evidence from experimental studies and systematic reviews indicates a range of self-reported mental health benefits from gardening-based activities and interventions, including reductions in depression, anxiety, stress, mood disturbance and loneliness, and improved quality of life, life satisfaction, cognition, positive relations with others and wellbeing (17–28). These findings are supported by physiological data indicating reductions in cortisol secretion and inflammation and maintained blood supply and neuroprotection to the brain following gardening based activities and interventions, all of which may lower the risk of psychiatric morbidities (25, 29). However, much of the literature has explored the effect of gardening interventions broadly including interventions that do not meet the criteria for STH and individuals with and without symptoms or diagnosis of a mental health condition (17–19, 25, 27–29). Furthermore, systematic reviews on STH have primarily focused on older adults (20–22), outcomes other than anxiety and depression (23), or a range of mental health outcomes, with limited evidence focused on specific conditions (24, 30, 31). Reviews also inconsistently apply STH criteria. For example one recent review of horticultural interventions that reported moderate-large effects for depression compared to usual care alone (32), did not exclusively include studies where trained practitioners administered the intervention or individuals with symptoms or diagnosis of depression, making it difficult to determine effectiveness. The lack of synthesised and accessible evidence of STH for specific conditions is a barrier to commissioning STH interventions (33). Thus, to reduce the strain on the NHS posed by increasing rates of depression and anxiety and continued shortages in support, and for STH to be commissioned more widely, evidence of the benefits for depression and anxiety is required. The aim of this systematic review and meta-analysis is to examine the effectiveness of STH for reducing symptoms of depression and anxiety in adults identified as at risk, with symptoms or with a diagnosis of depression and/or anxiety.

## Methodology

### Study registration

A systematic review was conducted in line with the protocol submitted to PROSPERO (Registration CRD42024542671) and following the Preferred Reporting Item for Systematic Reviews and Meta-analysis (PRISMA; See **Supplementary Figure S1**) (34). As this is a review of existing published literature, ethical approval and participant consent were not required.

### Inclusion and exclusion criteria

All quantitative study designs were eligible for inclusion including randomised controlled trials (RCT) and single group pre-post studies. Inclusion and exclusion criteria were formulated using the Population, Intervention, Comparator, and Outcome (PICO) approach (35):

Population:

Adults aged 18years+ who:

1. Have been identified as being at risk, having symptoms or have a diagnosis of depression and/or anxiety by a medical professional

2. Have below average scores, or scores outside of the ‘normal’ range on a validated measure of depression and/or anxiety (e.g. Depression, anxiety, and stress scale; see **Supplementary Table S3**).

3. Have been identified as having depression and/or anxiety through the use of a diagnostic scale (e.g. Generalised Anxiety Disorder- 7).

Intervention:

An STH intervention of any duration which:

1. Primarily focuses on horticultural activities (e.g., planting, potting, harvesting).

2. Is developed or led by trained practitioner(s) with experience and/or training in delivering social and therapeutic horticulture interventions (e.g., horticultural therapists).

3. Is conducted in any setting (e.g., community-based, hospital grounds) and environment (e.g., indoor, outdoor).

4. Can be in addition to treatment as usual, where treatment as usual is any treatment, intervention, or activity which might be used to address the primary health concern.

Comparator:

1. No treatment, those on a waiting list or receiving another type of intervention or treatment. Studies with no comparators were also included.

Outcome:

1. Scores for any type of depression and/or anxiety measured using validated scales or diagnostic tools.

### Search strategy

A search of the titles and abstracts of literature published in English language from 1973, the year that the American Therapeutic Horticulture Association was founded (or database inception), was conducted in PsychINFO, Medical Literature Analysis and Retrieval System Online (MEDLINE), Web of Science Social Sciences Citation Index (SSCI); Cochrane Central Register of Controlled Trials (CENTRAL), Cumulative Index to Nursing and Allied Health Literature (CINAHL), and Sports DISCUS. A search of university dissertations and theses was also conducted through EThOS and PROQuest. The search strategy included use of subject headings (including medical subject headings) and keywords and can be found in **Supplementary Table S2**. Keywords were co-produced with individuals with lived experience of depression and/or anxiety (n=4) and members of the Therapeutic Horticulture Stakeholder group (36). A grey literature search of the MIND and Mental Health Foundation websites was conducted via Google advanced search, alongside searches in The Kings Fund library database. Bibliographies of included studies and previous reviews were also hand searched, along with the Journal of Therapeutic Horticulture. All searches were conducted between May and June 2024.

### Eligibility screening and data extraction

References retrieved from the scientific databases were downloaded (n=1024) and exported into Rayyan Systematic Review software (37) where duplicates (n=243) were removed (see **Supplementary Figure S1**). References identified via other sources were recorded in an Excel spreadsheet (n=74). One reviewer (CJW) independently screened the titles and abstracts (n=855) against the pre-determined eligibility criteria. A second reviewer (CLW) screened a selection of articles from the scientific databases (n=78, 10% of scientific articles), with 100% agreement between reviewers. Eligible articles from scientific databases (n=77) and other sources (n=35) were retrieved for full text review. Two reviewers (CJW and CLW) independently screened the full texts, with any uncertainty discussed and a third independent reviewer (JB) sought where agreements were not possible.

For each study identified for inclusion, demographic details, a description of the intervention including the setting, activities and duration, information about the comparator group (where applicable), study design and the method of outcome measurement were entered into a data charting table. The mean and standard deviation of depression and/or anxiety scores at baseline and the first post-intervention timepoint were extracted for STH interventions and comparators. Data extraction commenced on 31/05/2024. Where more information was required on a study or data was not available, the study authors were contacted. If authors did not reply after two attempts at contact, articles without sufficient data or information were excluded.

### Risk of bias

Risk of bias for RCTs was assessed using the Joanna Briggs Institute Critical Appraisal tool for RCTs (38). This tool assesses each RCT against domains associated with bias in RCTs including 1. Selection and allocation of participants; 2. Administration of intervention; 3. Assessment, detection, and measurement of the outcome; 4. Participant retention and 5. Statistical conclusion validity. There was also one question about overall trial design. Studies were assessed as being either ‘low’, ‘unclear’ or ‘high’ risk of bias across each of these domains. Across all studies blinding of participants to the interventions was given a rating of ‘high’ risk as it is not possible to blind participants in this context.

Quasi-experimental studies including single group pre-post studies were assessed using the Joanna Briggs Institute Critical Appraisal tool for quasi-experimental studies (38). This tool assesses each study against domains associated with bias in quasi-experimental studies including 1. Temporal precedence; 2. Selection and allocation; 3. Confounding factors; 4. Administration of intervention; 5. Assessment, detection, and measurement of the outcome; 6. Participant retention; 7. Statistical conclusion validity.

All studies were independently rated by two authors (CJW and CLW). Following discussion around the interpretation and scoring of the RoB tools in relation to differences between groups at baseline and reliability of measurement of outcomes, 100% agreement between the two authors was reached. For all studies a points system was employed where studies were given one point for each question with a ‘low’ rating. For RCTs the overall score ranged from 0-13, whilst for quasi-experimental studies the overall score ranged from 0-9, with a higher score indicating a lower risk of bias.

### Data analysis

A narrative synthesis summarised effects for depression and anxiety across studies. Where available, statistical findings and effect sizes were used for comparison. A random effects meta-analysis was conducted using Review Manager version 5.3. As all studies used continuous outcomes but differing psychological constructs, standardised mean difference (SMD) was calculated. One meta-analysis was conducted for RCTs and quasi experimental studies with comparators, with the SMD being calculated by taking the mean of the intervention group from the mean of the comparator group, divided by the pooled standard deviation (SD). For anxiety, a sub-group comparison of RCTs and quasi-experimental studies with comparators was also reported. A second meta-analysis was conducted for single group pre-post studies where the mean post intervention score was subtracted from the mean pre-intervention score and divided by the pooled SD.

For all studies, the first post intervention measure was used as the comparison, except for Kam et al. (39) where only the mean change was available. In one study the comparison group were not eligible for inclusion in the review (40) due to anxiety scores within the normal range, this data was therefore treated as a single group study in the meta-analysis. In one study where both trait and state anxiety were measured (41) a combined score (in line with the scoring procedure for the construct) was used in the analysis. Where SDs were not available these were estimated using the calculation recommended by the Cochrane Handbook for systematic reviews of interventions (42). SMDs were interpreted using Cohen’s d, where d <0.2 was negligible, 0.2≤ d <0.5 small, 0.5≤ d <0.8 medium and ≥0.8 large (43). Negative effect sizes indicated that the STH intervention reduced symptoms of depression or anxiety.

Heterogeneity of intervention effects was investigated using the I^2^ statistic which represents the percentage of variability in a set of effect sizes due to between study variability (44). Values of 25%, 50% and 75% indicate low, moderate and high heterogeneity respectively (45). The chi-squared statistic was also considered with P values of ≤.10 indicating heterogeneity of the intervention effects (46).

Subgroup analyses were conducted to explore effects by outcome severity at baseline and intervention setting (e.g. indoors, outdoors) where data for at least two studies were available. For outcome severity, individual score ranges and normative values were used for each of the different outcome measures. Studies using measures without established cut points or normative values were not included in the sub-group analysis.

Publication bias via funnel plot was conducted for the meta-analysis on RCT and quasi experimental studies with comparators for depression. Publication bias was not explored for the remaining comparisons as the small number of studies per sub-group meant that these would be underpowered (47). Across all applicable analyses, statistics were recalculated and re-reported following the removal of one study (48) whereby the 95% confidence intervals (CI) of the estimated effect of the intervention did not cross with any other studies.

## Results

### Study design characteristics

The searches identified 17 unique studies for inclusion (**Supplementary Figure 1**). The key characteristics of the studies are presented in **Supplementary Table 2**. Studies included RCTs (n=4), quasi-experimental studies with comparators (n=10) and single group pre-post studies (n=3). Studies were primarily conducted in Korea (n=9) and the USA (n=2), with one study each in China, Sweden, Iran, Japan, Switzerland, and Taiwan. All studies were conducted between 2010 and 2023. Nine studies reported outcomes for depression only, two reported on anxiety only and six reported on both depression and anxiety. However, in one of these studies (49) depression scores were not eligible for inclusion and only anxiety was included.

### Participant characteristics

Across the studies the total number of participants was 879, with individual sample sizes ranging from 9 (50) to 291 (51). There was a wide age range of participants, with mean ages ranging from 32.1 years (40) to 89.0 years (52). In one study (51) the inclusion criteria were individuals aged over 13 years, however the average age for the sample was 53.48 years and this study was therefore included in the review. One other study focused on mothers and children (53), however only the data for mothers was included in the review. One study did not report the participants’ age (54).

Across the studies, five included females only (40, 41, 53, 55, 56), whilst the remainder included both male and female samples. Two studies did not report participants’ gender (54, 57), however Verra et al. (57) did include gender as a covariate in the analysis, indicating that the sample was mixed. Twelve studies focused on samples who did not require a mental health diagnosis, symptoms, or risk to be included in the study. The sample populations across these twelve studies were varied, with two focusing on stroke patients (49, 58), two on older adults in care facilities (52, 59) and one on older adults in a homeless facility (60), carers of elderly with dementia (55), mothers with children (53), pre-menopausal women (40), married middle aged women (41), individuals with chronic back pain (57), released prisoners (61), and female immigrants (56). Four of these studies had exclusion criteria focused on mental health with two excluding individuals with severe psychiatric conditions (57, 59), one excluding people requiring hospitalisation or medication for depression or anxiety (41), and one people with chronic conditions affecting activities of daily living or requiring prescribed medication (40). One further study focused on stroke patients but also had inclusion criteria that required participants to have symptoms of depression (48). Four studies were specifically focused on individuals with diagnosis or symptoms of a mental health condition, including adults with a diagnosis of schizophrenia, bipolar or major depressive disorder (39), adult outpatients with chronic depression (54), military veterans with at least one mental health diagnosis (50) and individuals aged 13+years with mild depressive or anxiety symptoms.

### STH settings and activities

Interventions were conducted in a variety of settings. Seven of the interventions were exclusively conducted indoors including in health or care facilities (48, 52, 55, 56, 59, 61) and a botanical garden greenhouse (40). Five studies were conducted in outdoor garden settings, one of which was in a hospital garden (54) and four of which were community-based garden settings including university campuses (39, 49, 50, 53). One study involved a mixture of different outdoor settings which were in both community and health or care settings (51). Three studies involved the use of both indoor and outdoor settings at a hospital (58), homeless living facility (60) and pain programme therapy garden and greenhouse (57). One study did not explicitly report the intervention setting (41).

The activities performed as part of the interventions included sowing seeds, potting, digging, weeding, fertilising, flower arrangement and bouquet creation. Activities varied depending on the cultural contexts of the interventions and whether they were aligned with any therapeutic or rehabilitation programmes relevant to the population group such as in the cases of several studies (41, 48, 52, 55, 56, 58, 60). Other studies reported specific objectives across their STH sessions which informed the activities delivered (39, 40, 49–51, 53, 59). Intervention length varied from four (55, 57, 61) to sixteen weeks (60), with intervention frequency varying from once per week (48, 52, 53, 60, 61) to five times per week (39). Intervention duration ranged from 30-40 minutes (52) to 3.5 hours (49). Eleven of the interventions were developed or led by horticultural therapists (40, 41, 50, 51, 53–55, 57–60). The remainder were developed or led by professionals such as nurses and occupational therapists who had training or experience in STH (39, 48, 52, 56, 61), or by a team with horticultural and therapeutic qualifications, training, or experience (49).

### Comparator groups

Out of the 17 studies included in the review, three did not include comparators (50, 53, 61). The remaining studies all included comparators, however information on the comparators varied across studies. The majority of studies had a comparator that was treatment as usual (TAU) for the condition being addressed, including regular sheltered workshop training (39), occupational therapy (48), standard stroke rehabilitation/care (49, 58), routine care (52), normal daily activities (51, 54), rational emotional behavioural therapy (56), or usual pain management (57). In these cases the intervention group also received treatment as usual. Four studies specified that they had a control but did not provide any further information (41, 55, 59, 60), and one study had an art group as a comparator (40), but this was not eligible for inclusion in the review based on the participants pre-intervention anxiety scores.

### Outcome measures

Across the seventeen studies, seven different measures of depression and four different measures of anxiety were used. Five studies used scales that measured depression and anxiety simultaneously, with three (39, 50, 54) using the Depression, Anxiety and Stress Scale (62–66) and two (49, 57) using the Hospital Anxiety and Depression Scale (67). However, in one of these studies depression scores were not eligible for inclusion in the review (49). One further study (51) measured both depression and anxiety, using the mental health screening tool for depressive disorders (68) and the mental health screening tool for anxiety disorders (69).

The remaining studies only measured either depression or anxiety. The Geritatric Depression Scale (70) was used in one study (48), with the short form (71–73) being used in a further four studies (52, 58–60) and in multiple lanugages. Two studies (55, 61) also measured depression using the Centre for Epidemiological Studies Depression Scale (74, 75) and two (53, 56) using the Korean version of Beck’s Depresion Inventory Short Form (76, 77). The only remaining measure of anxiety was the state-trait anxiety inventory (78), which was used in two studies (40, 41).

### Assessment of risk of bias

#### Randomised controlled trials

One study complied with 7/13 (54%) items, two studies complied with 6/13 (46%) items and one study complied with 3/13 (23%). The main limitations related to blinding of participants and intervention facilitators, and incomplete follow-up (e.g. not all participants included in analyses). Three studies did not evidence appropriate statistical analyses and were likely underpowered to detect statistically significant differences (**Supplementary Figure 2**). All studies were judged to be “unclear” regarding whether outcomes were measured in a reliable way, as the experience of the person administering measures was omitted from the paper along with information on how, where and when the measure was administered, and whether this was in line with the guidelines for the specific measure.

#### Quasi-experimental studies

Of the thirteen quasi experimental studies (n=3 single group studies and n=10 studies with comparators) four studies complied with 5/9 items (56%), two studies complied with 4/9 items (44%), six studies complied with 3/9 (33%), and one study complied with 2/9 items (22%). The main limitations related to lack of multiple measurements both pre and post intervention and low statistical conclusion validity (**Supplementary Figure 3**). All studies were judged to be “unclear” regarding whether outcomes were measured in a reliable way.

### Narrative summary

#### Depression

Fourteen of the seventeen studies reported on eligible depression outcomes. Only one of these studies was a RCT (39), ten were quasi experimental studies with comparators (48, 51, 52, 54–60) and three were single group pre-post studies (50, 53, 61). The findings of the RCT (39) revealed that the reduction in symptoms of depression was statistically greater in the intervention group, with the average score in the STH group moving from a ‘moderate’ to ‘normal’ range by the end of the STH intervention (**Supplementary Table 3**).

Across the quasi-experimental studies with comparators, findings were largely in favour of the STH interventions. Several studies demonstrated statistically greater reductions in depression in the STH group compared to the comparator group (51, 52, 54, 59), with one study reporting an effect size of 0.58 (51). Other studies using only within group comparisons revealed statistically significant reductions in depression in the STH group and no statistically significant changes in the comparator group (58), or statistically significant reductions in the STH group and statistically significant deteriorations in depression scores in the comparator group (56). Kim (48) reported statistically significant reductions in depression scores in both the STH and comparator group, however this reduction was larger in the STH group. Across the quasi-experimental studies with comparators the STH intervention average depression scores moved from a ‘severe’ to ‘moderate’ score range (54), ‘moderate’ to ‘normal’ score range (51, 52, 58) or ‘mild’ depression, or potential symptoms of depression to ‘normal’ range (48, 56) by the end of the intervention.

Two quasi-experimental studies reported no statistically significant differences in post-intervention depression scores and no statistical changes over time in either the STH or comparator group (55, 60). Verra et al. (57) reported no statistically significant differences between the STH and comparator group, who undertook the usual pain management programme, but reported a statistically significant reduction in depression in the STH group and no statistically significant change in the comparator, with effect sizes of 0.36 and 0.15 respectively. The three single group pre-post studies all revealed statistically significant reductions in depression scores following STH (50, 53, 61), with scores moving from being classified as ‘mild’ depression, or potential symptoms of depression at baseline to ‘normal’ at follow-up. One quasi-experimental study reporting no statistically significant changes in depression also found that depression scores moved from a ‘mild’ to ‘normal’ range by the end of the intervention (60), whilst Verra et al. (57) found a shift from a ‘moderate’ to ‘mild’ score following the STH intervention.

#### Anxiety

Eight of the seventeen studies reported on anxiety outcomes (see **Supplementary Table 2**). Four of these studies were RCTs (39–41, 49), three were quasi experimental studies with comparators (51, 54, 57) and one was a single group pre-post study (50). Amongst the RCTs, one study reported a statistically greater reduction in anxiety in the STH group compared to the comparator where anxiety increased (39). Another study using only within group comparisons reported a statistically significant reduction in state, trait and total anxiety in the STH group but not in the comparator condition (41). By contrast one study (49) reported no significant differences in the change in anxiety between groups, with both groups reducing their scores over time. The final study (40) examined state and trait anxiety in both the STH and a comparator group (art-making), however only the trait-anxiety scores in the STH group were eligible for inclusion in the review, with the study reporting a statistically significant reduction in trait anxiety in the STH group with an effect size of -0.47. Across all studies, participants in the STH intervention moved from a score indicative of anxiety at baseline to a score within ‘normal’ range at follow-up (40, 41, 49), with mean anxiety scores in STH participants in the study of Kam and Siu (39) moving from ‘severe’ to ‘normal’ range.

All three quasi-experimental studies revealed greater reductions in anxiety in the STH group compared to the comparator group, where there were no statistically significant changes in anxiety (51, 54, 57). Yang et al. (51) reported a large effect of 0.73 with STH participants anxiety moving from ‘mild’ to ‘normal’ range, whilst Verra (57) reported a small effect of 0.23 with scores in the STH group moving from a ‘moderate’ to ‘mild’ range. In the remaining study (54) anxiety scores moved from the ‘extremely severe’ to ‘severe’ range in the STH group. The only single group pre-post study focused on anxiety did not report any statistically significant changes, however, anxiety scores moved from the ‘moderate’ to ‘normal’ range by the end of the intervention (50).

### Meta-analysis

#### Depression

**Figure 1:**
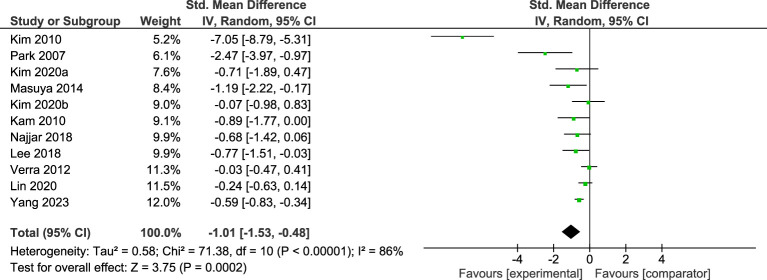
Meta-analysis of STH for depression vs comparator at post-intervention. The size of the green box reflects how much weight each study received in the meta-analysis. Black bars represent the 95% CI for the SMD in each study. CI, confidence interval; SMD, standardised mean difference.

Eleven studies including RCTs and quasi experimental studies with comparators, representing a total of 662 participants, were included in the meta-analysis. **Figure 1** demonstrates that across the 11 studies there was a large and significant effect in favour of reductions in depression in the STH group. High levels of heterogeneity were observed across the pooled analysis (I^2^ = 87%, *p*<.001), with the funnel plot (**Supplementary Figure 4**) indicating asymmetry and with one of the eleven studies (48) having a mean effects size outside of the 95%CI of the SMD. When this study, which focuses on stroke patients with mild depression, was removed from the analysis the effect was moderate and significant (-.55, p<.001) with moderate heterogeneity (I^2^ = 48%, p=.04).

**Figure 2:**
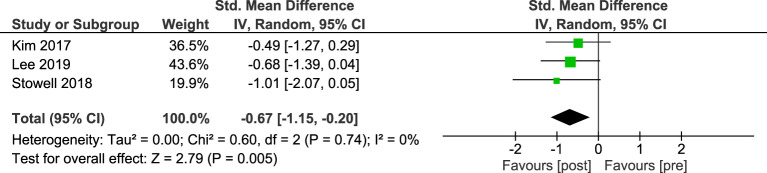
Meta-analysis of STH for depression in pre-post studies without comparators. The size of the green box reflects how much weight each study received in the meta-analysis. Black bars represent the 95% CI for the SMD in each study. CI, confidence interval; SMD, standardised mean difference.

A second meta-analysis was conducted for the three single group pre-post intervention studies which included a total of 37 participants. A moderate and significant effect in favour of post-intervention depression scores was observed (**Figure 2**). No heterogeneity was detected across the pooled analysis (I^2^ = 0%, p=.74).

Sub-group analysis comparing studies where participants started with mild or moderate-severe depression scores revealed a large and significant effect across studies where participants had scores indicative of mild depression and a small-moderate and significant effect across studies where participants had moderate-to severe depression (**Supplementary Figure 5**). There was significant high heterogeneity in studies focused on mild depression (I^2^ = 93%, p<.001) with the two studies included in this comparison (48, 56) having the largest effect sizes across all depression studies. There was low heterogeneity across studies focused on moderate-severe depression (I^2^ = 29%, p=.19) and no significant difference between sub-groups (χ^2^ (1) = 3.43; p=.06).

Additional subgroup analysis on studies that took place indoors compared to outdoors or a mixture of indoors and outdoors revealed a large and significant effect in interventions conducted indoors compared to a moderate and significant effect for those conducted outdoors or a mixture (**Supplementary Figure 6).** There was high heterogeneity amongst indoor studies (I^2^ = 94%, p<.001), but low heterogeneity in outdoor and mixed studies (I^2^ = 22%, p=.27). When Kim et al. (48), a study whereby the 95% CI of the estimated effect of the intervention did not cross with any other studies, was removed from the indoor studies the effect was large but non-significant (-.79, p=.06) with moderate heterogeneity (I^2^ = 72%, p=.01). There was no significant difference between sub-groups (χ^2^ (1) = 3.09; p=.08).

#### Anxiety

**Figure 3:**
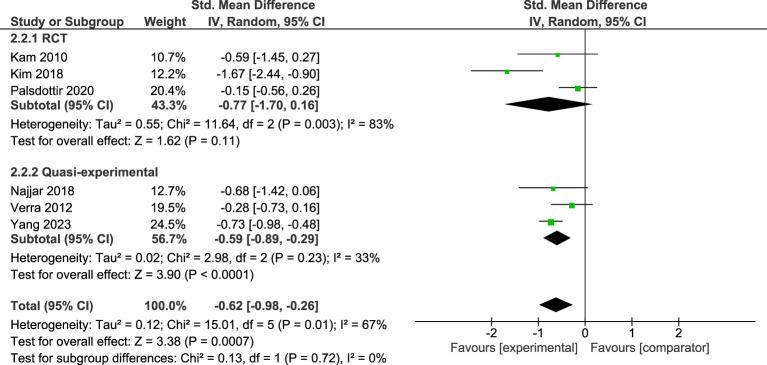
Meta-analysis of STH for anxiety vs comparator at post-intervention. The size of the green box reflects how much weight each study received in the meta-analysis. Black bars represent the 95% CI for the SMD in each study. CI, confidence interval; SMD, standardised mean difference.

Six studies with comparator groups, representing a total of 550 participants, were included in the meta-analysis. **Figure 3** demonstrates that across the six studies there was a moderate and significant effect in favour of reductions in anxiety in the STH group. Significant moderate heterogeneity was observed across the pooled analysis (I^2^ = 67%, p=.01). By intervention type, the findings revealed a moderate but not significant effect in favour of the experimental group in RCTs, with significant high heterogeneity (I^2^ = 83%, p=.003). Amongst quasi-experimental studies with comparators there was a moderate and significant effect in favour of the experimental group, with low heterogeneity (I^2^ = 33%, p=.23). There were no significant differences in the estimated effect between the two study types (χ^2^ (1) = .13; p=.72).

**Figure 4:**
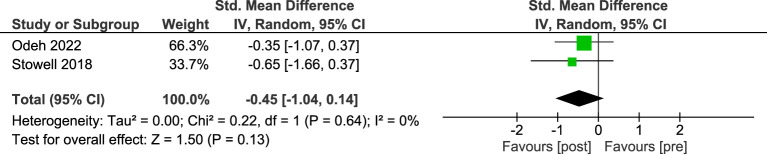
Meta-analysis of STH for depression in pre-post studies without comparators. The size of the green box reflects how much weight each study received in the meta-analysis. Black bars represent the 95% CI for the SMD in each study. CI, confidence interval; SMD, standardised mean difference.

Meta-analysis of the two single group pre-post studies, including 23 participants, found a small and non-significant effect in favour of post-intervention anxiety scores (**Figure 4**). No heterogeneity was detected across the pooled analysis (I^2^ = 0%, p=.64).

Comparison of the pooled effect of studies where participants started with mild anxiety compared to moderate-severe anxiety revealed small but not statistically significant effects for mild anxiety, compared to small and statistically significant effects for moderate-severe anxiety (**Supplementary Figure 7**). There was high heterogeneity in studies focused on mild anxiety (I^2^ = 82%, p=.02) but not moderate-severe (I^2^ = 0%, p=.61). There was no significant difference between sub-groups (χ^2^ (1) = .02; p=.89).

## Discussion

This systematic review and meta-analysis sought to examine the effectiveness of STH for reducing symptoms of depression and anxiety. Overall 17 unique studies were identified for inclusion in the review, 14 of which reported on depression outcomes and eight of which reported on anxiety outcomes. For both depression and anxiety, the narrative summary was largely in favour of STH interventions, with studies including comparators demonstrating greater reductions in depression and anxiety in the STH group. For depression, the single group pre-post studies also reported significant reductions in depressive symptoms at the post-intervention timepoint. The results of the meta-analysis supported these findings, with a large and significant effect in favour of reductions in depression in the STH group and a moderate and significant effect in favour of reductions in anxiety in the STH group across studies with comparators. For depression there was also a moderate and significant effect in favour of post-intervention scores for single pre-post studies.

These findings align with evidence supporting the beneficial effects of NBIs (79–82), gardening, community gardening and STH (18–20) for depression and anxiety outcomes across various clinical and non-clinical groups. This study is however, the first to present evidence of the effectiveness of STH interventions for reducing symptoms of depression and anxiety in adults with a diagnosis or symptoms of depression and anxiety. The findings highlight the potential of STH to supplement existing treatment approaches for depression and anxiety and provide evidence to support commissioning of STH interventions within mental healthcare. Given that the level of demand for mental health support is 2-3 times NHS capacity (10), STH interventions could provide an important means of reducing NHS waiting lists. STH interventions are also typically available for longer periods than treatments such as TTs and can be used flexibly through recovery and relapse (16, 83), thus they may also help to reduce the demand posed by pre-existing mental illness.

Along with demonstrating the beneficial impact of STH for depression and anxiety outcomes, sub-group meta-analysis revealed no significant differences in the impact of the interventions by outcome severity at baseline (mild vs moderate-severe) or intervention location (indoor vs outdoor/mixed). This evidence supports the use of STH across varying levels of mental health need, recovery, and relapse (16) and demonstrates the flexibility of such interventions across a range of settings. For example, in settings where it is not possible for participants to access outdoor environments, possibly due to ill-health, a lack of such spaces or bad weather, STH interventions can be adapted to indoor environments and can be used in community and clinical settings. However, these findings should be interpreted with caution as there were only a small number of studies per comparison for mental health severity and a comparison of intervention location was not possible for anxiety. There was also high heterogeneity across many comparisons, indicating variability in outcomes. Further research to explore the impact of intervention location and symptom severity at baseline is therefore required.

Unlike other medical treatment approaches for depression and anxiety, STH interventions improve multiple health outcomes simultaneously (22). Evidence suggests that gardening and STH interventions can improve physical health through increased levels of physical activity, reductions in body mass index and improved flexibility and endurance (21, 84–86). STH also improves psychosocial outcomes, reducing loneliness and isolation and increasing social interaction; and improves quality of life, life satisfaction and wellbeing, through increased feelings of meaning and purpose (20, 83, 86–92). STH interventions can also provide employment opportunities, through the development of horticulture skills, knowledge, and qualifications (83, 93). Thus, the potential of STH interventions for improving the health of the whole person is significant and use of such an intervention could result in substantial savings to the UK economy and NHS through improved health outcomes for individuals, reduced demands for services and treatment of physical, mental, and social comorbidity. Whilst several publications have demonstrated this point (91, 94), a cost-benefit analysis of a rigorously designed STH intervention for the treatment of depression and anxiety is yet to take place.

In the majority of studies included in the review, the inclusion criteria were not focused on mental health, despite participants having risk or symptoms of depression and/or anxiety at baseline. Only five of the seventeen studies required participants to have a mental health diagnosis for inclusion (39, 48, 50, 51, 54), with one of those being alongside a recent stroke (48). In several studies the primary focus was on rehabilitation or management of physical health conditions such as stroke (48, 49, 58), peri-menopause (40) and chronic back pain (57), reflecting its ability to target multiple health outcomes. Given that chronic physical health conditions are associated with mental ill-health and that individuals with mental ill-health often experience poorer physical health (95–98), the ability of STH to address multiple conditions simultaneously is of huge importance. In fact, mental ill-health, cardiovascular disease (including stroke) and musculoskeletal disorders are three of the six major health conditions that drive 60% of ill-health and early death in England (99). The remaining three conditions, which include cancers, dementia and chronic respiratory disease, could also be improved by STH, with published research already demonstrating the benefits of STH for individuals with dementia (100). Thus, STH interventions could have considerable impact across some of the costly health conditions to the UK economy and provide substantial benefits for the individual.

Aside from physical health conditions, the remaining studies in the review were focused on supporting individuals in adverse social circumstances, such as carers (55), those in care (52, 59) or homeless facilities (60), released prisoners (61), and female immigrants (56). Individuals from these groups are more likely to experience mental ill-health; for example, 80% of homeless people and 36% of prisoners are estimated to have a mental health condition, with homelessness and imprisonment often being the result of unresolved mental health inequalities (101–103). Individuals from these groups may also experience barriers to seeking and accessing mental health support, partially due to lack of coordination between health and social care services (103). STH could therefore help to address physical, mental, and social health inequalities and may be more accessible and acceptable to a range of different groups. However, further research on the acceptability and reach of STH interventions is required as many of the studies included in this review did not report on participant characteristics such as ethnicity or socio-economic status, two characteristics that influence access to mental health treatment (104), or whether participants self-selected to take part in the studies due to a prior interest in gardening. The previous UK Governments major conditions strategy case for change and strategic framework highlighted a commitment to tackling the wider determinants of health and to accelerating research to understand how mental, physical and social conditions interlink and can be treated (99). Although it is unclear how this commitment will be actioned under the new government; STH interventions can play a key role in addressing these major issues through the ability to tackle multiple issues concurrently.

Whilst evidence from this review highlights the potential of STH interventions to prevent and treat depression and anxiety, there are a number of limitations and future research needs. First, the overall quality of the studies was low, with high risk of bias across all study types, which significantly impacts the validity of the study findings. Only four of the included studies were RCTs, highlighting the challenges associated with designing and delivering high-quality trials within the context of STH. The inability to blind participants to STH interventions introduces the potential for both performance and detection bias. Many studies did not blind individuals collecting outcome measurements, had short-term and incomplete follow up data, were not adequately powered and failed to include multiple measures of outcomes both pre- and post- intervention. Future studies should therefore ensure full blinding of researchers not involved in the direct delivery of interventions to minimise detection bias relating to outcome measures. They should also seek to collect multiple measures of depression and/or anxiety, pre- and post- intervention, with longer term follow-up periods of at least 12 weeks. Studies should also ensure that they are adequately powered and account for dropout of participants across the study period and control for these in the statistical analysis.

The systematic review and meta-analysis also highlighted considerable heterogeneity between studies indicating variation in study outcomes. In the case of depression this was typically driven by one study (48) with effect sizes outside the 95%CI of the SMD. This study focused on stroke patients with depression and was unique in that its inclusion criteria required a physical illness alongside symptoms of depression. The remaining studies only had inclusion criteria focused on one primary physical, mental, or social condition, with the minority focusing on mental health. This makes it difficult to determine how the STH provisions across studies aligned with the identified levels of STH for mental health (16) and further influences the identified heterogeneity. In addition, the interventions were delivered across multiple countries where cultural differences may have influenced intervention delivery, engagement, and outcome. For example, Korea, where nine of the studies were conducted, have established regulatory bodies for STH and a rich history of gardening as part of their culture (105). Across studies the intervention location, frequency and duration were also highly varied, which may have further contributed to the identified heterogeneity. This variation also makes it difficult to determine whether there is an optimal intervention frequency and duration for individuals with symptoms of depression and anxiety.

In addition to the low quality of the studies and variation in study outcomes, which are likely due to flaws in experimental designs, no studies included in the review were conducted in the UK, with the majority being outside of Europe. Mental health was also not the primary outcome of concern in most of the studies, meaning that the “treatment as usual” comparator was often for a physical or social health concern. None of the comparators included typical medical treatment options for depression and anxiety such as TTs or medication. This impacts the strength of the conclusions, as it is not possible to comment on the effectiveness of STH compared to treatments or interventions targeting depression, anxiety, or even mental health as primary health conditions. Given that the NHS and the UK Government have committed to the use of NBIs for mental ill-health (12), and that STH interventions align with the Government’s community-based mental health model (13) and a whole-systems approach to healthcare (14), rigorous RCTs exploring the effectiveness of STH interventions compared to treatment as usual for depression and anxiety and in a UK context should be prioritised. However, the design of any randomised trial in this field is not without its challenges, and in particular the ethical issues associated with the randomisation of individuals with mental ill-health will need careful consideration in trial design to ensure all individuals receive timely adequate mental health support.

Across all studies, the most common reason for exclusion from this review was that the intervention did not meet the criteria for STH. The criteria employed in this study required interventions to be developed or led by trained practitioners with experience and/or training in delivering STH interventions. Some studies were excluded as a result of not providing sufficient information to determine whether this criterion was met and not responding to author queries to request this information. Thus, some eligible studies may not have been included in the review. Conversely, many studies did not meet the criteria but identified their interventions as STH, TH or HT. This ‘improper’ use of terminology confuses the evidence base and makes it difficult to distinguish between STH interventions and self-directed or social gardening activities. Whilst some countries such as the United States and Korea have regulatory bodies for STH, this is not consistent across the globe. In the UK there is currently no professional body dedicated to STH, a factor that may explain the lack of UK studies. However, Trellis and Thrive are currently working collaboratively to develop the UK Association for STH (106, 107), which will be accredited by the Professional Standards Authority and hold a register of STH practitioners who meet agreed national standards. It is hoped that this association will help to formalise the sector and further develop STH in the UK. The accreditation can also be used alongside the Green Care Quality Mark (108) which enables STH providers to demonstrate that they operate a safe, and professional organisation and meet expected standards by referral agencies, commissioners, and service users. However, currently only 110 organisations are involved in the Green Care Quality Mark scheme, and some work is required to grow this further. Alongside the growth of these associations, it would also be useful to explore the active mechanisms of STH interventions for depression and anxiety to inform intervention delivery guidelines. It is currently unknown whether the various components of STH work in combination to improve health outcomes or whether specific aspects drive improvements, such as exposure to nature, social support and interaction, or mastery of new skills.

Overall, the findings of the systematic review and meta-analysis highlight the potential of STH interventions to support existing mental health treatment approaches for depression and anxiety and reduce the strain on the NHS. The review also highlights the potential for interventions such as STH to help to tackle health inequalities and address physical, mental, and social conditions simultaneously. However, for the full potential of STH to be realised in the UK and to support upscaling of interventions, rigorous RCTs exploring the effectiveness of STH compared to “treatment as usual” for depression and anxiety are required. Greater regulation of the sector in the UK, evidence of the cost-effectiveness, and feasibility and acceptability of STH interventions for a wide range of groups are also required.
