# Supplementary material for: Effectiveness of social and therapeutic horticulture for reducing symptoms of depression and anxiety: a systematic review and meta-analysis
Source: Front Psychiatry. 2025 Jan 16;15:1507354. doi: 10.3389/fpsyt.2024.1507354 (PMC11799672; doi:10.3389/fpsyt.2024.1507354)
Supplement: Supplementary file 1 [file DataSheet1.docx]

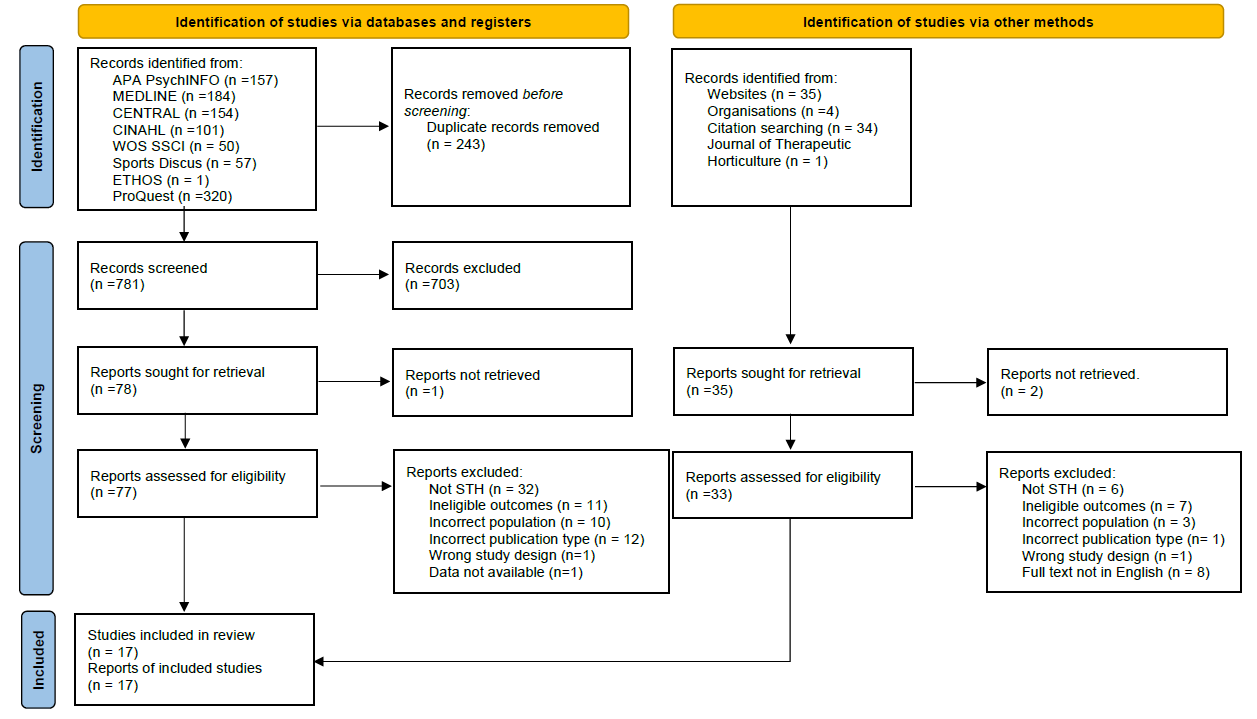


**Supplementary Figure 1: Prisma flow chart of study identification, inclusion, and exclusion.**

**Supplementary Table 1: Search Terms for each of the scientific databases**

| **Database** | | **Concept 1: Social and Therapeutic Horticulture** | **Concept 2: Depression and Anxiety** |
| --- | --- | --- | --- |
| PsychInfo | APA thesaurus | Horticulture therapy | Depression (emotion)  Major Depression  Anxiety  Anxiety Disorders |
|  | Title/Abstract | Social and Therapeutic Horticulture  Horticultural Therap*  Therapeutic Horticulture  Therapeutic Gardening  Therapeutic Community Gardening  Community Gardening  Gardening Therap*  Garden*  Horticultur* | Depression  Depressive disorder*  Depressive symptom*  Mood disorder*  Low mood  Depressed mood  Anxiety  Anxiety disorder*  Anxiety symptom*  Anxious  Generali#ed anxiety disorder  Mental health  Mental illness  Mental ill-health  Mental ill health  Mental disorder*  Mental wellbeing  Mental well-being |
| Medline | MESH terms | Horticulture (explode to include gardening)  Horticultural Therapy | Depression  Depressive Disorder (explode to inc Major Depressive Disorder)  Anxiety  Anxiety Disorders |
|  | Title/Abstract | Social and Therapeutic Horticulture  Horticultural Therapy*  Therapeutic Horticulture  Therapeutic Gardening  Therapeutic Community Gardening  Community Gardening  Gardening Therapy*  Garden*  Horticultur* | Depression  Depressive disorder*  Depressive symptom*  Mood disorder*  Low mood  Depressed mood  Anxiety  Anxiety disorder*  Anxiety symptom*  Anxious  Generalised anxiety disorder*  Mental health  Mental illness  Mental ill-health  Mental ill health  Mental disorder* |
| WoS ASSIA | N/A |  |  |
|  | Title/Abstract | Social and Therapeutic Horticulture  Horticultural Therapy*  Therapeutic Horticulture  Therapeutic Gardening  Therapeutic Community Gardening  Community Gardening  Gardening Therapy*  Garden*  Horticultur* | Depression  Depressive disorder*  Depressive symptom*  Mood disorder*  Low mood  Depressed mood  Anxiety  Anxiety disorder*  Anxiety symptom*  Anxious  Generalised anxiety disorder*  Mental health  Mental illness  Mental ill-health  Mental ill health  Mental disorder* |
| Central | MeSH | Horticulture (explode to include gardening)  Horticultural Therapy | Depression  Depressive Disorder (explode to inc Major Depressive Disorder)  Anxiety  Anxiety Disorders |
|  | Title/Abstract | Social and Therapeutic Horticulture  Horticultural Therapy*  Therapeutic Horticulture  Therapeutic Gardening  Therapeutic Community Gardening  Community Gardening  Gardening Therapy*  Garden*  Horticultur* | Depression  Depressive disorder*  Depressive symptom*  Mood disorder*  Low mood  Depressed mood  Anxiety  Anxiety disorder*  Anxiety symptom*  Anxious  Generalised anxiety disorder*  Mental health  Mental illness  Mental ill-health  Mental ill health  Mental disorder* |
| CIHNAL | CINAHL headings | Horticulture | Depression  Anxiety  Anxiety Disorders |
|  | Title/Abstract | Social and Therapeutic Horticulture  Horticultural Therapy*  Therapeutic Horticulture  Therapeutic Gardening  Therapeutic Community Gardening  Community Gardening  Gardening Therapy*  Garden*  Horticultur* | Depression  Depressive disorder*  Depressive symptom*  Mood disorder*  Low mood  Depressed mood  Anxiety  Anxiety disorder*  Anxiety symptom*  Anxious  Generalised anxiety disorder*  Mental health  Mental illness  Mental ill-health  Mental ill health  Mental disorder* |
| Sports Discus | Thesaurus | None | Mental depression  Anxiety |
|  | Title/Abstract | Social and Therapeutic Horticulture  Horticultural Therapy*  Therapeutic Horticulture  Therapeutic Gardening  Therapeutic Community Gardening  Community Gardening  Gardening Therapy*  Garden*  Horticultur* | Depression  Depressive disorder*  Depressive symptom*  Mood disorder*  Low mood  Depressed mood  Anxiety  Anxiety disorder*  Anxiety symptom*  Anxious  Generalised anxiety disorder*  Mental health  Mental illness  Mental ill-health  Mental ill health  Mental disorder* |
| Ethos | N/A |  |  |
|  | Title/Abstract | Social and Therapeutic Horticulture  Horticultural Therapy*  Therapeutic Horticulture  Therapeutic Gardening  Therapeutic Community Gardening  Community Gardening  Gardening Therapy*  Garden*  Horticultur* | Depression  Depressive disorder*  Depressive symptom*  Mood disorder*  Low mood  Depressed mood  Anxiety  Anxiety disorder*  Anxiety symptom*  Anxious  Generalised anxiety disorder*  Mental health  Mental illness  Mental ill-health  Mental ill health  Mental disorder* |
| ProQuest Dissertations and Theses |  | Horticulture  Gardening  Gardens and Gardening | Depression (psychology)  Anxiety  Anxiety Disorders |
|  | Title/Abstract | Social and Therapeutic Horticulture  Horticultural Therapy*  Therapeutic Horticulture  Therapeutic Gardening  Therapeutic Community Gardening  Community Gardening  Gardening Therapy*  Garden*  Horticultur* | Depression  Depressive disorder*  Depressive symptom*  Mood disorder*  Low mood  Depressed mood  Anxiety  Anxiety disorder*  Anxiety symptom*  Anxious  Generalised anxiety disorder*  Mental health  Mental illness  Mental ill-health  Mental ill health  Mental disorder* |

**Supplementary Table 2: Characteristics of included studies**

|  | | | **Participants** | | | | **Intervention** | | | | | | |
| --- | --- | --- | --- | --- | --- | --- | --- | --- | --- | --- | --- | --- | --- |
| **Author (year)** | **Country** | **Study type** | **Sample** | **No. (E/C)** | **Age (E/C)** | **Female (E/C)** | **Setting** | **Activities** | **Frequency** | **Duration (mins)** | **Facilitator(s)** | **Comparator** | **Measures**  **(D/A)** |
| Kam (2010) [39] | China | RCT | Diagnosis of schizophrenia spectrum, bipolar, or major depression disorder | 12  12 | 45.3±10.4  43.3±11.7 | 33.0%  25.0% | Outdoor gardens of New Life Farm | Watering & fertilising plants, weed removal, harvesting, scarecrow and herbal teabag making, potting plants. | Five weekly sessions for two weeks | 60 | Occupational Therapist with horticultural therapy training | Regular workshop training (TAU) | Depression, Anxiety and Stress Scale short form, Chinese version |
| Kim (2010) [48] | Korea | Quasi experimental with comparator | Stroke patients with hemiplegia hospitalised and scoring 20+ on mini mental state examination and 10+ on geriatric depression scale | 20  20 | 58.0  66.0 | 25%  45% | Indoors at hospital | Flower arrangements, sowing, creations using leaves, making pot pourri, vegetable planting | Four sessions per month for three months | Not reported | Occupational Therapists and Horticulturalists | Occupational therapy (TAU) | Geriatric Depression Scale |
| Kim (2017) [61] | Korea | Single group pre-post | Released prisoners | 13 | 48.9±10.8 | 7.7% | Indoors in rehabilitation agency | Creating bouquets, planting, and learning about plants, planting creation of garden | One weekly session for four weeks | 60 | Mental health nurse with horticultural therapy degree | None | Centre for Epidemiologic Studies-Depression Scale Korean version |
| Kim (2018) [41] | Korea | RCT | Married middle aged women not requiring hospital treatment or medication due to depression or anxiety | 18  18 | 40-59 | 100% | Not reported | Planting, flower arrangements, making crafts with plants | Two weekly sessions for twelve weeks | 60 | Two horticultural therapists | Inactive Control (no details provided) | State-trait anxiety inventory |
| Kim (2020a) [60] | Korea | Quasi experimental with comparator | Homeless elderly in homeless living facility | 6  6 | 74.0±5.1  72.4±5.5 | 33.3% | Garden at homeless facility and programme hall | Sowing seeds, harvesting, transplanting, setting up supports for plants, making bouquets, walking & making extracts/punch | One weekly session for sixteen weeks | 60-90 | Horticultural therapists | Inactive Control (no details provided) | Geriatric Depression Scale short form Korean Version |
| Kim (2020b) [55] | Korea | Quasi experimental with comparator | Carer of elderly with dementia | 15  15 | 58.8±9.6  61.3±12.4 | 100% | Multi-purpose program room in the health centre of Haman-gun. | Garden formation, growing, flowering, fruitation and harvest. | Two weekly sessions for four weeks | 90-120 | Organised by professor of horticultural therapy, psychiatry, and social welfare, and a PhD candidate in horticultural therapy. Carried out by one main and one assistant horticultural therapist | Inactive Control (no details provided; TAU) | Centre for Epidemiologic Studies-Depression Scale Korean version |
| Lee (2018) [58] | Korea | Quasi experimental with comparator | Stroke inpatients | 14  17 | 53.4±12.6  56.1±10.0 | 57.1%  41.2% | Occupational therapy room and rooftop garden of a hospital | Planting and transplanting plants, making flower garden beds | Three weekly sessions for eight weeks | 60 | Horticultural therapist | Standard stroke rehabilitation programme (TAU) | Geriatric Depression scale short form Korean Version |
| Lee (2019) [53] | Korea | Single group pre-post | Mothers with children <7years | 16 | 37.8±3.5 | 100% | Garden, green house and activity area of a care farm. | Making a plot, sowing seeds planting, harvesting, and cooking. | One weekly session for six weeks | 90 | Horticultural therapist | None | Beck Depression Inventory short form Korean version |
| Lin (2020) [59] | Taiwan | Quasi experimental with comparator | Older adults from two long-term care facilities | 59  59 | 77.4±7.5  78.4±6.9 | 19%  47% | In long term care facility | Plant familiarisation and cultivation, use of seeds to create puzzles, designing potted plants for relaxation. | Two weekly sessions for nine weeks | 90 | Two horticultural therapists | Inactive control (did not participate in any similar program) | Geriatric Depression scale short form Chinese version |
| Masuya (2014) [52] | Japan | Quasi experimental with comparator | Elderly residents of nursing homes aged 65+ with no diagnosis of depression, speech, or vision disorders | 9  9 | 89.0±7.1  82.2±6.6 | 77.8% | In a room separate to usual care setting | Growing, harvesting, pruning, fertilising. | One weekly session for six weeks | 30-40 | Dementia nurse with training in horticultural techniques | Routine care  (TAU) | Geriatric Depression Scale short form |
| Najjar (2018) [54] | Iran | Quasi experimental with comparator | Outpatients with chronic depression | 15  15 | Not reported | Not reported | Hospital garden | Planting, watering, weeding, picking flowers and creating flower boxes | Two weekly sessions for five weeks | 120 | Designed by horticultural therapist | Normal activities (TAU) | Depression, Anxiety and Stress Scale |
| Odeh (2022) [40] | USA | RCT | Pre-menopausal women aged 26-49yrs without chronic conditions | 20  20 | 32.1±5.1  32.8±5.6 | 100% | Greenhouse at Wilmot Botanical Gardens | Planting, propagation, transporting, harvesting plants and vegetables | Three weekly sessions for eight weeks | 85 | Registered horticulturalist therapist reviewed the activities. Horticulture student and study coordinator led activities. | Art (not eligible for inclusion in review) | State-trait anxiety inventory (only trait scores eligible for inclusion) |
| Palsdottir (2020) [49] | Sweden | RCT | Stroke patients aged 50-80yr | 50  51 | 67.0  66.0 | 53%  66% | Alnarp Rehabilitation Garden | Walking around the garden, gardening, and horticultural activities, harvesting. | Two weekly sessions for ten weeks | 210 | Occupational Therapist, Horticulturalist, Psychotherapist and Physiotherapist | Standard stroke care (TAU) | Hospital Anxiety and Depression Scale (only anxiety eligible for inclusion) |
| Park (2017) [56] | Korea | Quasi experimental with comparator | Female marriage immigrants | 7  7 | 41.3  41.7 | 100% | Group counselling office | Planting, flower arrangements, garden creation, plant topiary, rose soap creation | Ten sessions over approx. two months | 120 | Counselling leader with horticulture psychology qualification | Rational Emotional Behavioural Therapy (TAU) | Beck Depression Inventory short form Korean version |
| Stowell (2018) [50] | USA | Single group pre-post | Military veterans who self-reported at least one mental  health diagnosis. | 9 | 50.8±13.4 | 44.4% | Agricultural gardens of a university | Tilling, weeding, garden preparation and planting, garden design, watering, and care, propagating and tending | Two weekly sessions for five weeks | 90 | Horticultural Therapists | None | Depression, Anxiety and Stress Scale short form |
| Verra (2012) [57] | Sweden | Quasi experimental with comparator | Patients in pain programme who had suffered chronic non-specific back pain or fibromyalgia for at least 6 months | 41  47 | 49.0  47.1 | Not reported | Therapy garden and greenhouse | Theoretical information and learning about plants, sowing seeds, potting, vegetable gardening, digging, planting, bouquet creation. | Two weekly sessions for four weeks (7 sessions) | 60 | Horticultural therapist, physiotherapist, and horticulturalist | Usual pain management programme (TAU) | Hospital Anxiety and Depression Scales |
| Yang (2023) [51] | Korea | Quasi experimental with comparator | Aged 13+ years with mild depressive or anxiety symptoms | 192  99 | 52.2±24.0  56.0±24.0 | 75.5%  80.8% | Green areas nationwide | Planting, fertilizing, repotting, mulching, flower arrangement, and picnics in the garden. | Two weekly sessions for fifteen weeks | 120 | Clinical psychologist and horticultural therapist. | Daily activities provided by community centres participants were recruited from | Mental health screening tool for depressive disorders  Mental health screening tool for anxiety disorders |

E= Experimental Group; C= Comparator Group; D= Depression; A= Anxiety

**Supplementary Table 3: Mean±SD of pre- and post- intervention depression and anxiety outcomes for the STH and comparator groups.**

|  | | **Depression** | | **Anxiety** | | **SMD (95% CI)** | | **Measure, score range and normal values** |
| --- | --- | --- | --- | --- | --- | --- | --- | --- |
|  |  | **Pre** | **Post** | **Pre** | **Post** | **Depression** | **Anxiety** |  |
| **Kam (2010) [39]**  RCT | **STH** | 14.6±9.1 | -9.2±9.2* | 15.0±7.8 | -9.0±7.6* | -0.9  (-1.8, 0.0) | -0.6  (-1.5, 0.3) | Depression, Anxiety and Stress Scale short form Chinese version [62-66]  Range per sub-scale: 0-42  Normal score depression: 0-9  Normal score anxiety: 0-7 |
|  | **Comparator** | 9.3±8.9 | -1.2±8.3* | 9.8±8.7 | 0.7±7.1* |  |  |  |
| **Kim (2010) [48]**  Quasi experimental | **STH** | 11.6±1.5 | 6.0±0.8 |  |  | -7.1  (-8.8, -5.3) |  | Geriatric Depression Scale [70]  Range: 0-30  Normal score: 0-10 |
|  | **Comparator** | 16.9±1.7 | 15.1±1.6 |  |  |  |  |  |
| **Kim (2017) [61]**  Single group | **STH** | 17.4±14.6 | 11.2±9.5 |  |  | -0.5  (-1.3, 0.3) |  | Centre for Epidemiologic Studies Depression Scale: Korean version [74, 75]  Range: 0-60  Normal score: 0-15 |
| **Kim (2018) [41]**  RCT | **STH** |  |  | 84.1±19.6 | 62.8±12.9 |  | -1.7  (-2.4, -0.9) | State-Trait Anxiety Inventory [78]  Range for total score: 40-160  Range for each subscale: 20-80  Normal/low score: 20-39 |
|  | **Comparator** |  |  | 85.3±15.3 | 87.5±15.7 |  |  |  |
| **Kim (2020a) [60]**  Quasi experimental | **STH** | 5.8±3.1 | 4.8±2.4 |  |  | -0.7  (-1.9, 0.5) |  | Geriatric Depression Scale short form: Korean version [72]  Range: 0-15  Normal score: 0-5 |
|  | **Comparator** | 6.8±4.0 | 7.3±3.9 |  |  |  |  |  |
| **Kim (2020b) [55]**  Quasi experimental | **STH** | 38.5±11.8 | 35.9±11.8 |  |  | -.01  (-1.0, 0.8) |  | Centre for Epidemiologic Studies Depression Scale: Korean version [74,75]  Range: 0-60  Normal score: 0-15 |
|  | **Comparator** | 33.0±7.3 | 36.7±7.0 |  |  |  |  |  |
| **Lee (2018) [58]**  Quasi experimental | **STH** | 7.0±4.5 | 4.6±3.7 |  |  | -0.8  (-1.5, 0.0) |  | Geriatric Depression Scale short form: Korean version [72]  Range: 0-15  Normal score: 0-5 |
|  | **Comparator** | 6.5±3.3 | 7.4±3.4 |  |  |  |  |  |
| **Lee (2019) [53]**  Single group | **STH** | 11.3±6.7 | 6.3±7.7 |  |  | -0.7  (-1.4, 0.0) |  | Beck Depression Inventory (21 item): Korean version [76,77]  Range: 0-63  Normal score: 0-9 |
| **Lin (2020) [59]**  Quasi experimental | **STH** | 6.7 | 8.4±*3.4* |  |  | -0.2  (-0.6, 0.1) |  | Geriatric Depression scale short form: Chinese version (reverse scored) [71]  Range: 0-15  Normal score: 10-15 (reverse scoring) |
|  | **Comparator** | 7.5 | 7.6±*3.4* |  |  |  |  |  |
| **Masuya (2014) [52]**  Quasi experimental | **STH** | 5.7±2.8 | 3.6±2.2 |  |  | -1.2  (-2.2, -0.2) |  | Geriatric Depression Scale short form [73]  Range: 0-15  Normal score: 0-5 |
|  | **Comparator** | 6.4±3.6 | 6.9±3.0 |  |  |  |  |  |
| **Najjar (2018) [54]**  Quasi experimental | **STH** | 22.8±5.5 | 17.1±5.1 | 24.1±6.2 | 16.6±5.0 | -0.7  (-1.4, 0.1) | -0.7  (-1.4, 0.1) | Depression, Anxiety and Stress Scale [62-64, 66]  Range for each subscale: 0-42  Normal score depression: 0-9  Normal score anxiety: 0-7 |
|  | **Comparator** | 19.9±5.1 | 20.3±4.0 | 17.4±5.9 | 17.6±6.6 |  |  |  |
| **Odeh (2022) [40]**  RCT, only STH eligible | **STH** |  |  | 41.1±12.4 | 37.0±10.2 |  | -0.4  (-1.1, 0.4) | State-Trait Anxiety Inventory [78] (trait only)  Range for each subscale: 20-80  Normal/low score: 20-39 |
| **Palsdottir (2020) [49]**  RCT | **STH** |  |  | 7.6 | 6.3±*6.6* |  | -0.2  (-0.6, 0.3) | Hospital Anxiety and Depression Scale [67]: (anxiety only)  Range per sub-scale: 0-21  Normal score: 0-7 |
|  | **Comparator** |  |  | 7.9 | 7.4±*6.6* |  |  |  |
| **Park (2017) [56]**  Quasi experimental | **STH** | 10.6±2.5 | 4.1±1.8 |  |  | -2.5  (-4.0, -1.0) |  | Beck Depression Inventory (21-item): Korean version [76, 77]  Range: 0-63  Normal score: 0-9 |
|  | **Comparator** | 13.4±6.1 | 15.3±5.7 |  |  |  |  |  |
| **Stowell (2018) [50]**  Single group | **STH** | 11.8±6.0 | 5.5±5.7 | 10.6±5.3 | 7.0±5.3 | -1.0  (-2.1, 0.1) | -0.7  (-1.7, 0.4) | Depression, Anxiety and Stress Scale short form [62-64, 66]  Range per sub-scale: 0-42  Normal score depression: 0-9  Normal score anxiety: 0-7 |
| **Verra (2012) [57]**  Quasi experimental | **STH** | 48.5±21.8 | 56.4±23.1 | 48.8±23.7 | 55.0±20.3 | 0.0  (-0.5, 0.4) | -0.3  (-0.7, 0.2) | Hospital Anxiety and Depression Scale [67]  Range per sub-scale: 0-21 (authors converted to 0-100, with a lower score indicating less depression/anxiety)  Normal score: 0-7 (converts to 0-33) |
|  | **Comparator** | 52.4±22.0 | 55.7±24.2 | 48.1±22.5 | 48.6±24.0 |  |  |  |
| **Yang (2023) [51]**  Quasi experimental | **STH** | 17.3±9.5 | 8.4±9.4 | 16.9±8.7 | 8.5±9.6 | -0.6  (-0.8, -0.3) | -0.7  (-1.0, -0.5) | Mental Health Screening Tool for Depressive [68] and Anxiety [69] Disorders  Range: 0-48 depression, 0-44 anxiety  Normal score: 0-8 depression, 0-10 anxiety |
|  | **Comparator** | 18.0±10.7 | 14.2±10.6 | 17.8±10.1 | 15.7±10.4 |  |  |  |

A higher score indicates higher levels of depression and/or anxiety unless otherwise reported. *indicates reported post value was a change score, with a negative score indicating a reduction in depression or anxiety. Baseline score was based on all participants including two who had dropped out of the comparator by the follow-up. Italics indicates that the standard deviation was estimated for the meta-analysis.

Data to determine a ‘normal’ score range is derived from either the publication itself or published criteria for the scale (if available). Scores that were borderline were included in the review, with each individual article being evaluated against all inclusion criteria to establish eligibility.

| Bias related to | Selection and allocation | | | Administration of the intervention/exposure | | | Assessment, detection and measurement of the outcome | | | Participant retention | Statistical Validity | | Overall |  |  |
| --- | --- | --- | --- | --- | --- | --- | --- | --- | --- | --- | --- | --- | --- | --- | --- |
|  | D1 | D2 | D3 | D4 | D5 | D6 | D7 | D8 | D9 | D10 | D11 | D12 | D13 | Total | % |
| Kam (2010) |  |  |  |  |  |  |  |  |  |  |  |  |  | 6 | 46 |
| Kim (2018) |  |  |  |  |  |  |  |  |  |  |  |  |  | 3 | 23 |
| Odeh (2022) |  |  |  |  |  |  |  |  |  |  |  |  |  | 6 | 46 |
| Palsdottir (2015) |  |  |  |  |  |  |  |  |  |  |  |  |  | 7 | 54 |
| Note.  D1. Was true randomization used for assignment of participants to treatment groups?  D2. Was allocation to treatment groups concealed?  D3. Were participants included in any comparisons similar?  D4. Were participants blind to treatment assignment?  D5. Were those delivering the treatment blind to treatment assignment?  D6. Were treatment groups treated identically other than the intervention of interest?  D7. Were outcome assessors blind to treatment assignment?  D8. Were outcomes measured in the same way for treatment groups?  D9. Were outcomes measured in a reliable way?  D10. Was follow-up complete and, if not, were differences between groups in terms of their follow-up adequately described and analysed?  D11. Were participants analysed in the groups to which they were randomized?  D12. Was appropriate statistical analysis used?  D13. Was the trial design appropriate and any deviations from the standard RCT design accounted for in the conduct and analysis of the trial? | | | | | | | | | | | | | | \| Judgement \| \| \| \| --- \| --- \| --- \| \|  \| Low \| \|  \| Unclear \| \|  \| High \| | |

**Supplementary Figure 2: Risk of bias outcomes for randomised controlled trials**

| Bias relating to | Temporal precedence | Selection and allocation | Confounding factors | Administration of intervention/exposure | Assessment, detection and measurement of the outcome | | | Participant retention | Statistical Conclusion validity |  |  |
| --- | --- | --- | --- | --- | --- | --- | --- | --- | --- | --- | --- |
|  | D1 | D2 | D3 | D4 | D5 | D6 | D7 | D8 | D9 | Total | % |
| Kim (2010) |  |  |  |  |  |  |  |  |  | 5 | 56 |
| Kim (2017) |  |  |  |  |  |  |  |  |  | 3 | 33 |
| Kim (2020a) |  |  |  |  |  |  |  |  |  | 5 | 56 |
| Kim (2020b) |  |  |  |  |  |  |  |  |  | 3 | 33 |
| Lee (2018) |  |  |  |  |  |  |  |  |  | 3 | 33 |
| Lee (2019) |  |  |  |  |  |  |  |  |  | 3 | 33 |
| Lin (2020) |  |  |  |  |  |  |  |  |  | 2 | 22 |
| Najjar (2018) |  |  |  |  |  |  |  |  |  | 3 | 33 |
| Masuya (2014) |  |  |  |  |  |  |  |  |  | 5 | 56 |
| Park (2017) |  |  |  |  |  |  |  |  |  | 5 | 56 |
| Stowell (2018) |  |  |  |  |  |  |  |  |  | 3 | 33 |
| Verra (2012) |  |  |  |  |  |  |  |  |  | 4 | 44 |
| Yang (2023) |  |  |  |  |  |  |  |  |  | 4 | 44 |
| Note.  D1. Is it clear in the study what is the “cause” and what is the “effect”?  D2. Was there a control group?  D3. Were participants included in any comparisons similar?  D4. Were the participants included in any comparisons receiving similar treatment/care, other than the exposure or intervention of interest?  D5. Were there multiple measurements of the outcome, both pre and post the intervention/exposure?  D6. Were the outcomes of participants included in any comparisons measured in the same way?  D7. Were outcomes measured in a reliable way?  D8. Was follow-up complete and if not, were differences between groups in terms of their follow-up adequately described and analysed?  D9. Was appropriate statistical analysis used? | | | | | | | | | | \| Judgement \| \| \| --- \| --- \| \|  \| Low \| \|  \| Unclear \| \|  \| High \| \|  \| N/A \| | |

**Supplementary Figure 3: Risk of bias outcomes for quasi experimental studies**


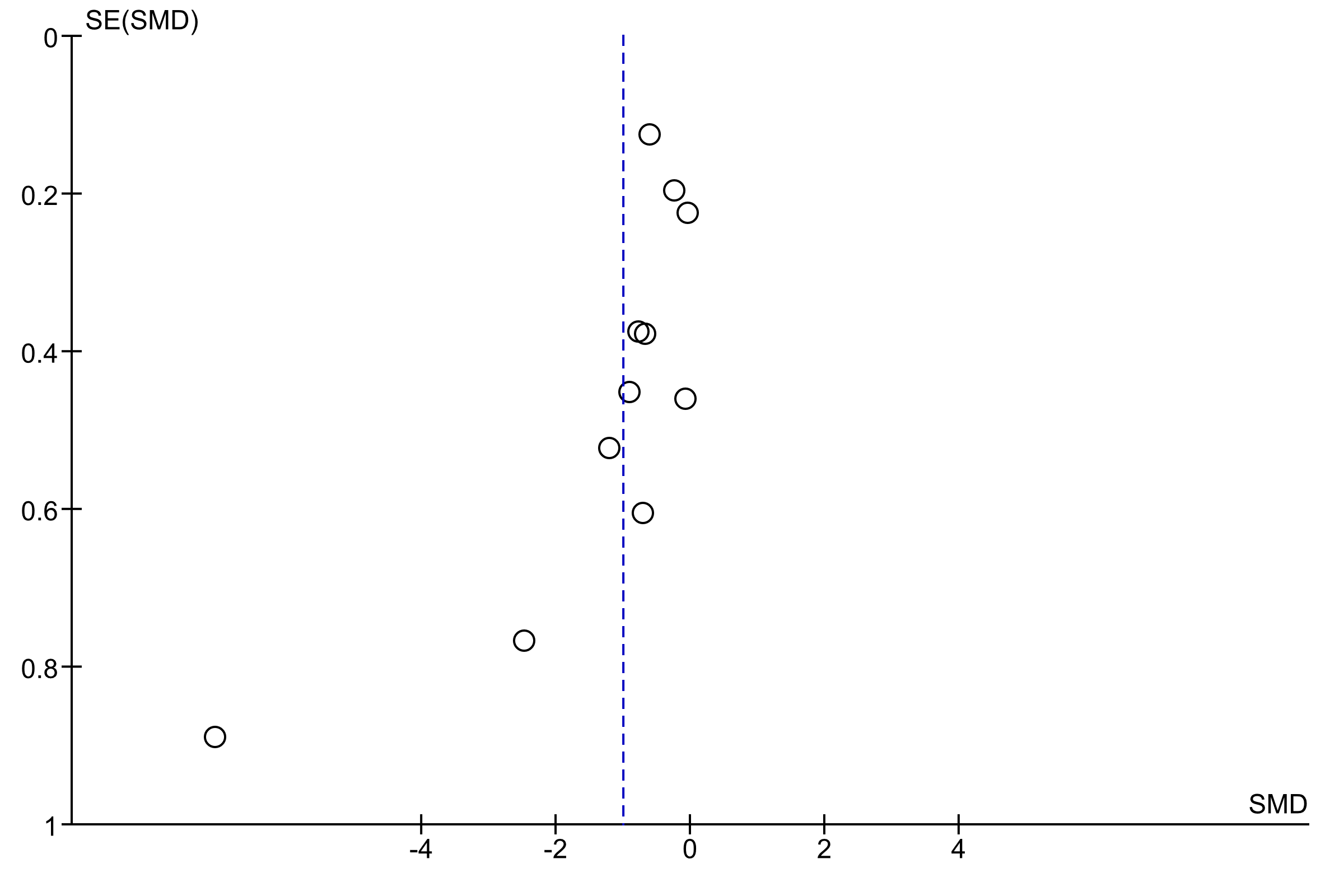


**Supplementary Figure 4: Funnel plot for STH for randomised controlled trials and quasi-experimental controlled studies**


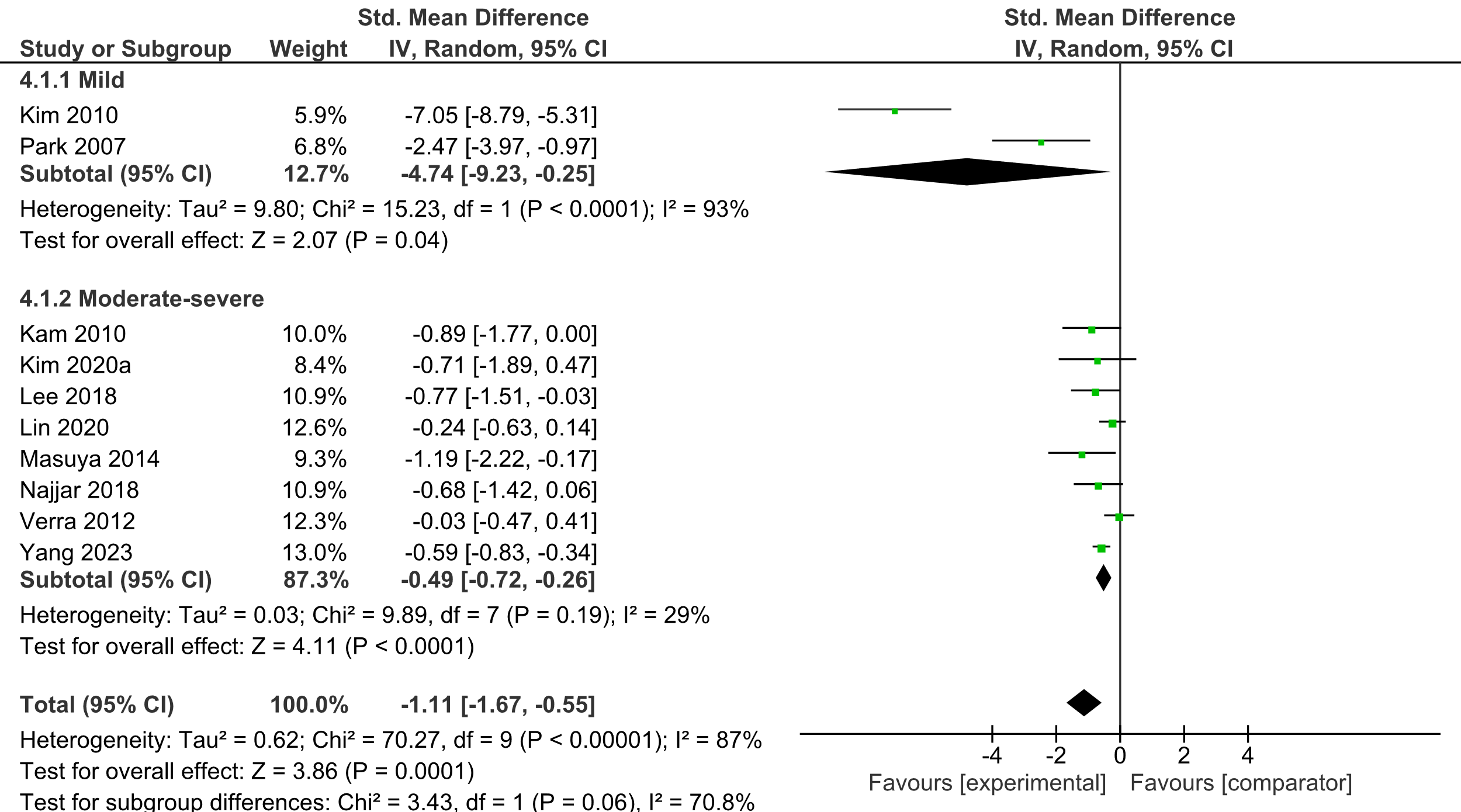


**Supplementary Figure 5: Meta-analysis of STH for depression vs comparator by severity of depression.** Note: The size of the green box reflects how much weight each study received in the meta-analysis. Black bars represent the 95% CI for the SMD in each study. CI= confidence interval, SMD= standardised mean difference.


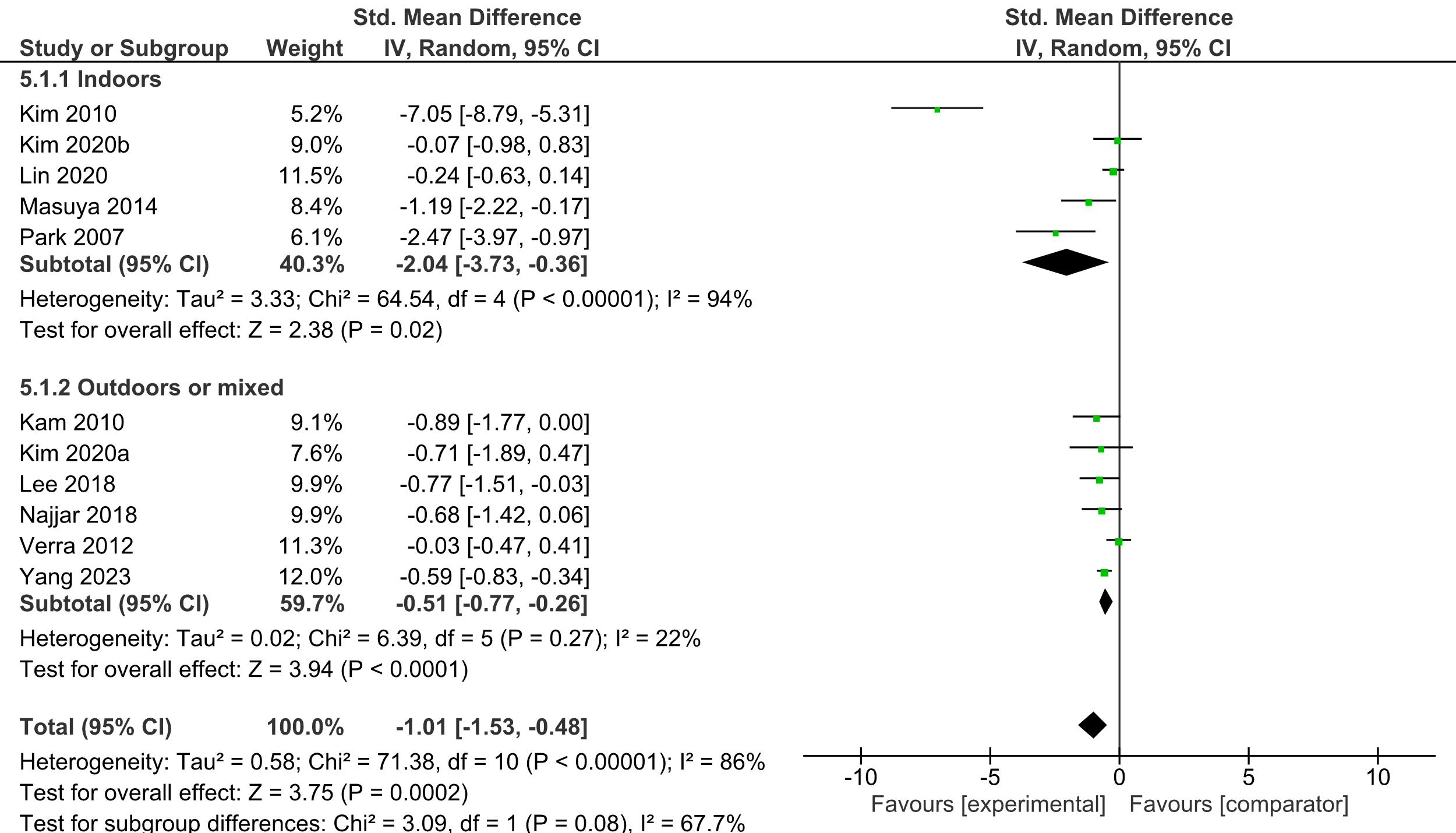


**Supplementary Figure 6: Meta-analysis of STH for depression vs comparator by intervention location.** Note: The size of the green box reflects how much weight each study received in the meta-analysis. Black bars represent the 95% CI for the SMD in each study. CI= confidence interval, SMD= standardised mean difference.


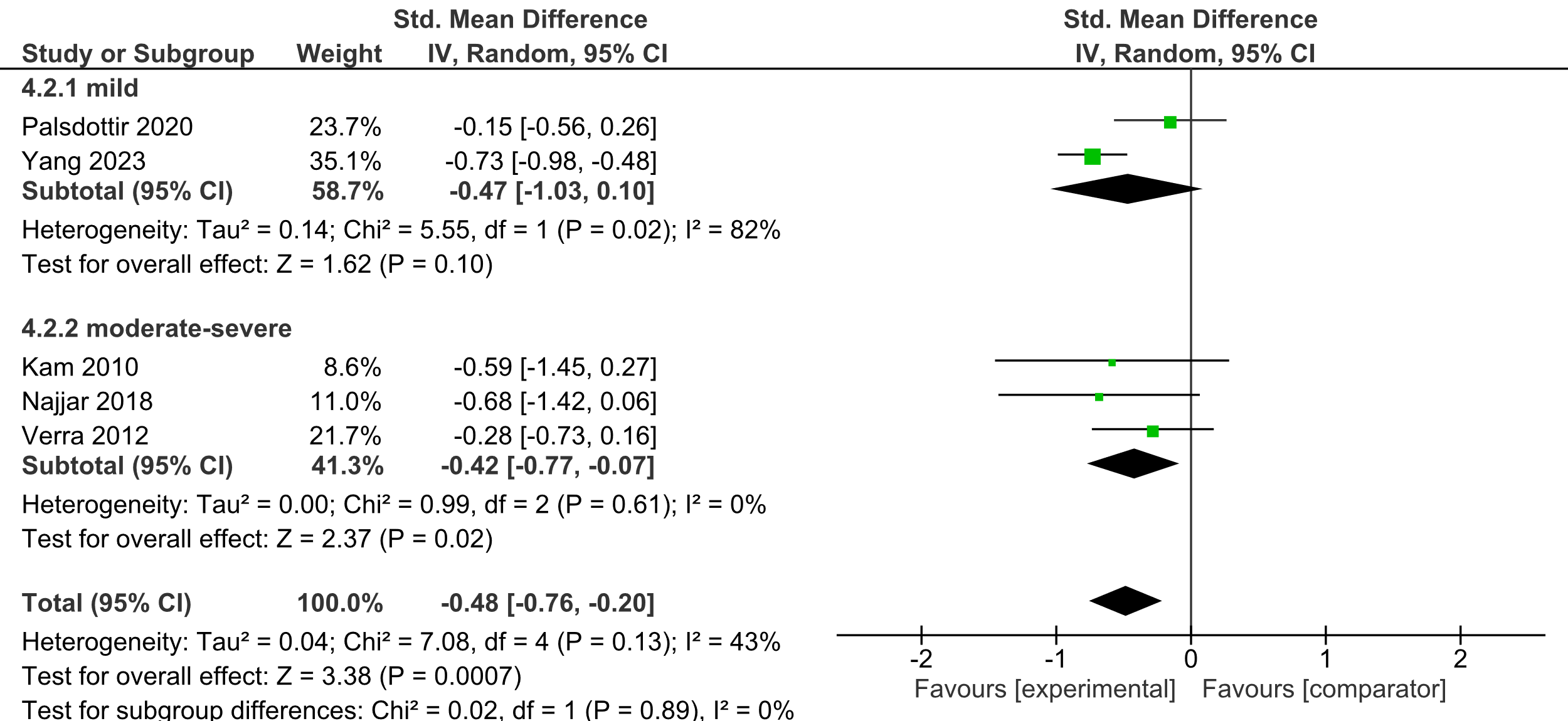


**Supplementary Figure 7: Meta-analysis of STH for anxiety vs comparator by severity of anxiety.** Note: The size of the green box reflects how much weight each study received in the meta-analysis. Black bars represent the 95% CI for the SMD in each study. CI= confidence interval, SMD= standardised mean difference.
